# Safety and efficacy of a new thromboprophylaxis regiment for total knee and total hip replacement: a retrospective cohort study in 265 patients

**DOI:** 10.1186/s13037-018-0169-x

**Published:** 2018-08-14

**Authors:** Mohammad Amre Fallaha, Sarkhell Radha, Sheena Patel

**Affiliations:** 1 Imperial College School of Medicine, London, UK; 2 Department of Orthopaedics, Chelsea and Westminster Hospital (CWH), London, UK; 3 Pharmacy Department, Chelsea and Westminster Hospital (CWH), London, UK

**Keywords:** Thromboprophylaxis, Rivaroxaban, Enoxaparin, Arthroplasty, Venous thromboembolism, Bleeding

## Abstract

**Background:**

Venous thromboembolism (VTE) remains a significant complication following knee and hip arthroplasty. National and international guidelines recommend pharmacological and mechanical thromboprophylaxis following surgery, unless contraindicated, to reduce the risk of VTE. This study aimed to explore the safety and efficacy profile of an adapted thromboprophylaxis regimen consisting of sequential enoxaparin and rivaroxaban for thromboprophylaxis following knee or hip arthroplasty at a London teaching hospital.

**Methods:**

A total of 265 patients who received sequential enoxaparin and rivaroxaban and mechanical thromboprophylaxis following knee and hip arthroplasty were included in the study. Efficacy outcomes assessed for 90 days post-operatively included: pulmonary embolism, deep-vein thrombosis, other VTE, myocardial infarction, stroke and death secondary to thrombosis. Safety outcomes were assessed during and for two days after thromboprophylaxis course duration and consisted of major bleeding episodes, clinically-relevant non-major bleeding episodes, and total bleeding.

**Results:**

There was 1 patient (0.4%) who experienced a stroke, and no other efficacy outcomes occurred. Major bleeding occurred in 2.3% (*n* = 6/265) of patients, whilst clinically-relevant non-major bleeding occurred in 3.4% (*n* = 9/265), with a total bleeding incidence of 16.2% (*n* = 43/265). No patients required a return to theatre.

**Conclusion:**

The regimen consisting of sequential enoxaparin and rivaroxaban is associated with a significant bleeding risk, although the risk of patients requiring a return to theatre is low. Further prospective trials are required to compare the safety and efficacy profiles of this regimen with established thromboprophylaxis regimens.

## Background

Venous thromboembolism (VTE), which encompasses deep venous thrombosis (DVT) and pulmonary embolism (PE), is a risk following all types of surgery, but is particularly significant following arthroplasty due to factors such as surgical trauma to vessels and prolonged immobility. The incidence of DVT in patients without thromboprophylaxis ranges between 40 and 60% [1].

The US-based American College of Chest Physicians (ACCP), in addition to the UK-based National Institute of Clinical Excellence (NICE) both recommend pharmacological and mechanical thromboprophylaxis to reduce the risk of VTE [2, 3]. The direct oral Factor Xa inhibitor, rivaroxaban (Xarelto®), has been included amongst those drugs recommended for thromboprophylaxis by the ACCP and NICE following the publication of the RECORD studies (The Regulation of Coagulation in Orthopaedic Surgery to Prevent Deep Vein Thrombosis and Pulmonary Embolism). The RECORD studies were a collection of four multicentre randomised-controlled trials which compared the safety and efficacy of rivaroxaban thromboprophylaxis against enoxaparin thromboprophylaxis [4–7]. These trials demonstrated superior efficacy of rivaroxaban in VTE event prevention compared to enoxaparin (Clexane®). However, concerns remain within the orthopaedic community regarding the safety profile (in particular, bleeding events) associated with rivaroxaban [8–10].

In response to local audit findings, local hospital guidelines recommend a sequential enoxaparin and rivaroxaban regimen as pharmacological thromboprophylaxis management in adult patients following knee or hip arthroplasty. Enoxaparin (via subcutaneous administration) is commenced post-operatively and continued during the inpatient stay, and then switched to rivaroxaban (via oral administration) on discharge for the appropriate duration, with either a total of 14 days thromboprophylaxis for knee arthroplasty or 35 days thromboprophylaxis for hip arthroplasty surgery. This regimen combines the safety of enoxaparin during the inpatient setting with the convenience of oral, rather than injectable, administration of rivaroxaban on discharge. In addition, mechanical thromboprophylaxis is offered for the duration of the inpatient stay following clinical assessment of contraindications during admission.

There has been a recent interest in this modified regimen [11, 12], but as far as the authors are aware, there have been no studies characterising the efficacy and safety of the adapted sequential enoxaparin and rivaroxaban regimen in both the inpatient and outpatient post-operative period following hip or knee arthroplasty. This exploratory single-centre study aimed to characterise the efficacy and safety of this regimen as thromboprophylaxis following knee and hip arthroplasty in our local cohort. In addition, we sought to determine whether there was an association between patient demographic information and bleeding events.

## Methods

A single centre, non-randomised outcome study was performed at the teaching hospital. All patients undergoing knee or hip arthroplasty between 1st January 2016 and 31st December 2016 inclusive were identified by procedure coding data. All patients over 18 years old who had undergone knee or hip arthroplasty and who were placed on sequential enoxaparin and rivaroxaban thromboprophylaxis were included on an intention-to-treat basis. Patients were excluded if the indication for arthroplasty was radiologically-confirmed hip fracture.

Patients received tranexamic acid intraoperatively and were commenced on enoxaparin 40 mg 6–12 h post-operatively, which was administered once daily for the duration of the inpatient admission. This was switched to rivaroxaban 10 mg once daily on discharge for the appropriate duration according to procedure type (see *Introduction*). Patients were offered and prescribed anti-embolism stockings during the inpatient admission.

Data were recorded prospectively at the time of patient admission using standardised formats, with retrospective data collection and analysis. A VTE risk assessment, assessing factors relating to thrombosis risk as well as bleeding risk, was completed for all patients on admission based on Trust guidelines. Demographic data were collected from the hospital’s electronic prescribing system, which included information such as height, weight, co-morbidities, medications and surgery details.

**Fig. 1:**
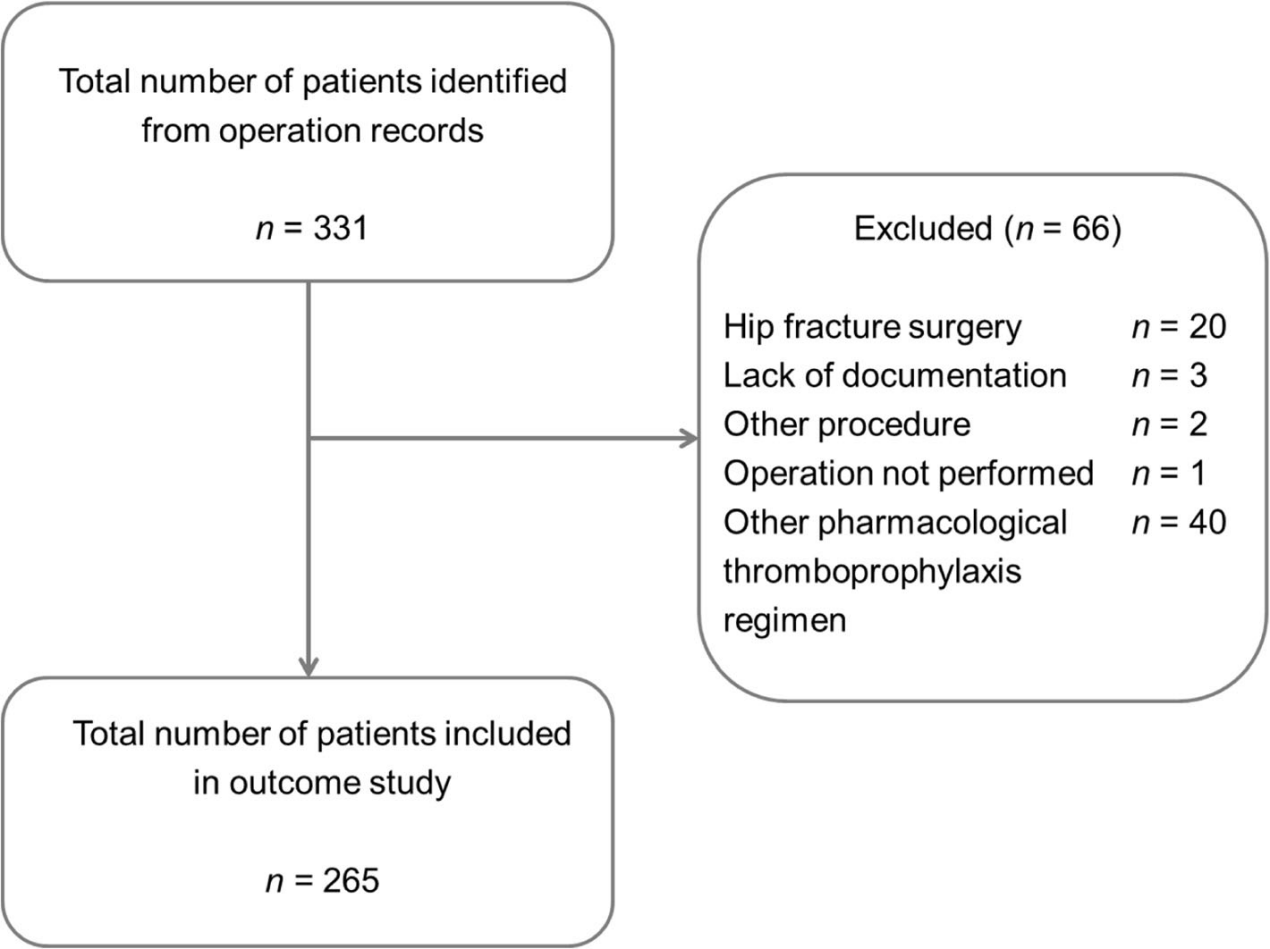
Patient inclusion and exclusion criteria

A total of 331 patients were identified from operation records and screened for inclusion, with 265 patients included in this study (Fig. 1). Other forms of pharmacological thromboprophylaxis that were excluded from this study were rivaroxaban only (*n* = 6) enoxaparin only (*n* = 24), use of apixaban (*n* = 3), altered doses of rivaroxaban and enoxaparin (*n* = 3), use of warfarin (*n* = 3), and continuation of rivaroxaban beyond 35 days post-operatively (*n* = 1). There was no loss to follow-up, and baseline patient data are presented in Table 1. Intra-operative anaesthetic notes were only available for 255 patients, so the length of operation was missing in 10 patients.

**Table 1:** Baseline patient demographics

	Procedure type		
	Hip (*n* = 151)	Knee (*n* = 114)	Total (*n* = 265)
Female - no (%)	88 (58.2)	80 (70.4)	169 (63.5)
Age (years)			
Mean	66	70	68
Range	32–91	47–90	32–91
Height (cm)			
Mean	167.9	163.2	165.8
Range	131.0–193.0	143.0–187.0	131.0–193.0
Weight (kg)			
Mean	79.4	80.8	80.0
Range	44.7–120.5	45.4–140.0	44.7–140.0
BMI (kg/m^2^)			
Mean	28.1	30.2	29.0
Range	17.2–46.2	19.7–47.9	17.2–47.9
Operation type - no (%)			
Primary	146 (96.7)	106 (93.0)	253 (95.1)
Revision	5 (3.3)	8 (7.0)	13 (4.9)
Fixation technique			
Cemented	35 (23.2)	110 (96.5)	146 (54.9)
Hybrid	50 (33.1)	0 (0.0)	50 (18.8)
Uncemented	66 (43.7)	3 (2.6)	69 (25.9)
Unknown	0 (0.0)	1 (0.9)	1 (0.4)
Operation duration			
Mean	1.9	2.2	2.03
Range	1.0–2.8	0.5–6.5	1.0–6.5
Comorbidities			
Chronic kidney disease	3 (2.0)	2 (1.8)	5 (1.9)
Hepatic failure	1 (0.7)	2 (1.8)	3 (1.1)
Diabetes Mellitus	12 (7.9)	17 (15.7)	29 (10.9)
IHD	8 (5.3)	8 (7.0)	16 (6.0)
Asthma/COPD	22 (14.6)	16 (13.9)	38 (14.3)
Hypertension	49 (32.5)	64 (55.7)	113 (42.6)
Atrial Fibrillation	5 (3.3)	4 (3.5)	9 (3.4)
Previous VTE	2 (1.3)	3 (3.5)	6 (2.3)
Vascular disease	2 (1.3)	1 (0.9)	3 (1.1)
Congestive Cardiac Failure	0 (0.0)	0 (0.0)	0 (0.0)
Total duration of prophylaxis (days)			
Mean	31–45	6–33	
Range	31–45	6–33	

Nine primary outcomes were assessed such that six outcomes (imaging-confirmed PE, imaging-confirmed DVT, other VTE, myocardial infarction, stroke, and death secondary to thrombosis) were used to assess the efficacy of pharmacological thromboprophylaxis efficacy, and three outcomes (major bleeding episodes, clinically-relevant non-major bleeding episodes, and total bleeding episodes) were used to assess safety profiles. Bleeding definitions were in accordance with the Scientific Standardisation Committee of the International Society of Thrombosis and Haemostasis [13, 14].

Major bleeding was defined as fatal bleeding, symptomatic bleeding in a critical area or organ, extrasurgical site bleeding causing a fall in haemoglobin of 20 g/L or more or requiring transfusion of two or more units of whole blood or red cells, or surgical site bleeding that required secondary intervention or was large enough to cause haemodynamic instability [13]. Criteria for a clinically-relevant non-major bleeding episode was any sign or symptom of haemorrhage that required medical intervention by an healthcare professional, resulted in an escalation of level of care, or prompted a face-to-face evaluation [14]. Other bleeding encompassed all other bleeding events, including from the wound. Superficial bleeding, or oozing, was defined as mild bleeding arising from the wound that was deemed prolonged by the assessing surgeon and subsequently documented in the notes as “oozing”.

Data collection and analysis involved review of patient medical notes and electronic records, Emergency Department admission notes and clinic correspondence. Operation date was defined as day 1, and patients were followed up for a period of 90 days after surgery, as root-cause analysis for hospital associated VTE events is performed for events occurring up to 90 days following recent admission [15]. Efficacy outcomes were included if the event occurred within 90 days of surgery, whilst safety outcomes were included whilst patients were on thromboprophylaxis and up to 2 days after completing thromboprophylaxis course, as per the RECORD trials [4–7].

### Statistical analysis

Statistical analysis was performed using SPSS Statistics (version 24, IBM Corporation) [16]. Univariate analysis was performed between categorical variables (gender, comorbidities, operation site, operation type, concomitant medication) and outcomes using the Chi-Squared Test or Fisher’s Exact Test where appropriate. Operation type encompassed both the site of the operation as well as whether the operation was a primary or revision procedure. The Student T-Test was used to compare the mean values of continuous variables (age, length of operation) and bleeding outcomes. A *P*_α_ value of 0.05 was initially set for significance, but to protect against a Type I error, a Bonferroni correction was applied such that the altered *P*_α_ = 0.002. Therefore, results were deemed significant if *p* ≤ 0.002.

## Results

The sequential enoxaparin and rivaroxaban regimen consisted of enoxaparin administration for patients during their postoperative inpatient stay, which was switched to rivaroxaban upon discharge. The average number of doses of enoxaparin for knee and hip arthroplasty patients was 3.7 (range 1–10) and 4.2 (range 1–23) respectively. 157 patients (59.2%) received antiembolism stockings, and 3 patients (1.1%) received intermittent pneumatic compression devices.

A non-haemorrhagic stroke was experienced by one patient (0.4%) and no other efficacy outcomes occurred. In addition to no deaths secondary to thrombosis occurring, there were no deaths reported in our patient cohort for the study duration. Bleeding events were experienced by 16.2% (*n* = 43/265) of patients, with an incidence of 21.0% and 12.6% in knee and hip arthroplasty respectively (chi-squared *p* = 0.064) (Table 2). This was not associated with the number of bleeding risk factors identified pre-operatively (Student t-test *p* = 0.346). Major bleeding events occurred in 2.3% (*n* = 6/265) of patients, whilst clinically-relevant non-major bleeding occurred in 3.4% of patients (*n* = 9/265).

**Table 2:** Postoperative complications

*n* (%)			Hip (*n* = 151)	Knee (*n* = 114)	Total (*n* = 265)
Any bleeding			19 (12.6)	24 (21.1)	43 (16.2)
Major Bleeding			3 (2.0)	3 (2.6)	6 (2.3)
Clinically relevant non-major bleeding			4 (2.6)	5 (4.4)	9 (3.4)
Other non-major bleeding			17 (11.3)	23 (20.2)	40 (15.1)
Wound complications					
	Haemorrhagic wound complications				
		Superficial bleeding	12 (7.9)	19 (16.7)	31 (11.7)
		Haematoma	8 (5.3)	2 (1.8)	10 (3.8)
	Joint effusion		0 (0.0)	7 (6.1)	7 (2.6)
	Wound infection		6 (4.0)	1 (0.9)	7 (2.6)

The most frequent wound complication reported was superficial bleeding, which was present in 11.7% (*n* = 31/265) of patients. Other wound complications included haematoma, joint effusion and wound infection, which occurred in 3.8% (*n* = 10/265), 2.6% (*n* = 7/265) and 2.6% (*n* = 7/265) of patients respectively. Of the 7 patients with a wound infection, 5 experienced a superficial wound infection only, 1 patient had a wound infection associated with a haematoma, and one patient had associated oozing from the wound site. All of these 7 patients were treated with a short course of antibiotics alone, and did not require surgical intervention.

Pharmacological thromboprophylaxis was required to be held in 4.1% (*n* = 11/265) of patients, and no patients in our cohort required a return to theatre. Blood transfusion(s) was required in 6.0% (*n* = 16/265) of patients.

There were no significant associations when baseline patient factors were analysed for association with all bleeding events (Table 3). When analysis for an association between all bleeding events was performed between primary and revision procedures, a *p* value of 0.042 was obtained. However, this remained insignificant as a result of the Bonferroni correction applied to our P_α_ value.

**Table 3:** Association of patient factors with bleeding events

*n* (%)^a^	Any bleed (*n* = 43)	No bleed (*n* = 222)	*p*
Female sex	28 (65.1)	140 (63.1)	0.798
Age (mean)	69.1	67.2	0.279
Renal failure	1 (2.3)	4 (1.8)	0.590
Hepatic failure	0 (0.0)	3 (1.4)	1.000
Diabetes	6 (14.0)	23 (10.4)	0.436
Ischaemic Heart Disease	0 (0.0)	16 (7.2)	0.083
Asthma and COPD	10 (23.3)	28 (12.6)	0.068
Hypertension	23 (53.5)	90 (40.5)	0.116
Atrial Fibrillation	3 (7.0)	6 (2.7)	0.165
Previous VTE	2 (4.7)	3 (1.4)	0.187
Vascular disease	0 (0.0)	4 (1.8)	1.000
Partial joint replacement	1 (2.3)	7 (3.2)	1.000
Hip replacement	19 (44.2)	132 (59.4)	0.064
Primary replacement	38 (88.3)	214 (96.4)	0.042
ACE inhibitor/ARB	15 (34.9)	62 (27.9)	0.358
Statin	11 (25.6)	75 (33.8)	0.293
Gastric Protection	16 (37.2)	75 (33.8)	0.665
CCB	10 (23.3)	51 (23.0)	0.968
Aspirin	1 (2.3)	11 (5.0)	0.697
Clopidogrel	1 (2.3)	2 (0.9)	0.413
Ticagrelor	0 (0.0)	0 (0.0)	–
Dihydropyridine	0 (0.0)	0 (0.0)	–
Diuretic	9 (20.9)	28 (12.6)	0.150
Antiretroviral	1 (2.3)	2 (0.9)	0.413
Warfarin	0 (0.0)	0 (0.0)	–
Length of operation (mean)^b^	2.16	2.02	0.188

^a^Unless specified otherwise

^b^Documentation only available for 255 patients (43 patients experienced any bleeding event and 212 patients did not experience any bleeding event)

*ACEi – Angiotensin converting enzyme inhibitor; ARB – Aldosterone receptor blocker; CCB – Calcium channel blocker; COPD – chronic obstructive pulmonary disease*

## Discussion

This study was, to the authors’ knowledge, the first attempt in the UK to describe the safety and efficacy profile of this adapted sequential enoxaparin and rivaroxaban regimen in hip and knee arthroplasty patients during admission and following discharge.

There was one patient who experienced a stroke, with no other efficacy outcomes experienced in the cohort study. The American College of Chest Physicians estimates an average on-prophylaxis rate of symptomatic DVT in orthopaedic surgery patients of 0.8% [3]. A cohort of 125 patients would therefore be expected to detect 1 symptomatic DVT event. However, although there was only one patient who experienced a non-haemorrhagic stroke with no other efficacy outcomes reported, this study was underpowered to determine the efficacy of the sequential enoxaparin and rivaroxaban regimen in preventing VTE. Therefore, no conclusions can be made regarding the efficacy of this regimen.

The observed total bleeding incidence of 16.2% in the present cohort study is substantially higher than the incidence reported in previous clinical trials with enoxaparin or rivaroxaban as thromboprophylaxis regimens following elective arthroplasty [4–7]. This incidence of total bleeding events ranged from 4.8% for enoxaparin 40 mg once daily, to 10.5% for rivaroxaban 10 mg once daily in the RECORD studies [5, 7]. Whilst this may suggest the regimen consisting of sequential enoxaparin and rivaroxaban possesses a greater bleeding risk, other local factors such as surgical technique, may also explain and contribute to the increased bleeding incidence in the present small cohort study.

A recent study directly compared a similar sequential administration of enoxaparin and rivaroxaban in patients undergoing internal fixation following hip fracture surgery, and demonstrated there was no difference in bleeding events compared to rivaroxaban alone, or enoxaparin alone [12]. A lack of control group in our study precludes a direct comparison with other thromboprophylaxis regimens, so further studies comparing the safety of this regimen to established thromboprophylaxis regimens following elective arthroplasty are required to adequately compare and contrast the safety of sequential enoxaparin and rivaroxaban in comparison to other thromboprophylaxis regimens.

Despite the high bleeding incidence in the present cohort study, no patients required further surgical intervention, which compares favourably to other studies which have reported an incidence of return to theatre due to wound complications of up to 3.9% with rivaroxaban alone [8, 9]. In addition, the present cohort study experienced a required blood transfusion rate of 6.0%, which is similar to the results found in a previously published similar cohort of patients receiving thromboprophylaxis following lower-limb arthroplasty, where the required transfusion rate was between 6.4 and 7.1% [8]. However it should be noted that blood transfusion practice may vary between hospitals. Finally, the incidence of major bleeding in the cohort study is similar to incidences reported in other studies [17–20].

### Limitations

As with all studies, this cohort study was subject to a number of limitations. Patient adherence was not assessed, so conclusions are made based on medicines prescribed and do not necessarily reflect medicine adherence. In addition, as the cohort study was conducted at a single site, it is not possible to conclude whether bleeding rates for the sequential enoxaparin and rivaroxaban thromboprophylaxis can be generalised, or are influenced by local hospital practice, surgical techniques and patient demographics.

The results must also be interpreted with caution given the relatively small sample size, and studies with larger cohorts are required to more accurately characterise the safety and efficacy of the proposed regimen. Our small sample size also limits the strength of our assessment of factors associated with bleeding. Furthermore, a lack of control group in the cohort study prevents direct comparison with other thromboprophylaxis regimens. Finally, unlike previous interventional studies, routine venography was not performed, so the detection of asymptomatic VTE events was not possible, but the clinical relevance of asymptomatic PE has been called into question previously [21]. Therefore, routine venography of patients in similar studies may not necessarily be required.

## Conclusions

In our small cohort of patients receiving sequential enoxaparin and rivaroxaban for pharmaceutical thromboprophylaxis in combination with mechanical thromboprophylaxis, bleeding events were common and patients receiving this regimen should be adequately counselled about this bleeding risk. It is unclear presently, based on the available literature and results from this study, how the incidence of bleeding events from this regimen compares to other thromboprophylaxis regimens. Prospective larger trials with control groups are required to adequately assess the safety and efficacy of the sequential enoxaparin and rivaroxaban regimen against established thromboprophylaxis regimens, such as enoxaparin and rivaroxaban alone.

## Abbreviations

DVT: Deep vein thrombosis
PE: Pulmonary embolism
VTE: Venous thromboembolism
